# *In situ* phytoremediation of copper and cadmium in a co-contaminated soil and its biological and physical effects

**DOI:** 10.1039/c8ra07645f

**Published:** 2019-01-09

**Authors:** Lei Xu, Xiangyu Xing, Jiani Liang, Jianbiao Peng, Jing Zhou

**Affiliations:** College of Environmental Science and Tourism, NanYang Normal University NanYang 473061 China; Key Laboratory of Soil Environment and Pollution Remediation, Institute of Soil Science, Chinese Academy of Science Nanjing 210008 China zhoujing@issas.ac.cn +86-13913387498; Henan Key Laboratory of Ecological Security for Water Source Region of Mid-line of South-to-North Diversion Project NanYang 473061 China; College of Non-Major Foreign Language Teaching, Nanyang Normal University 473061 Nanyang China; School of Environment, Henan Normal University Xinxiang 453007 China; Jiangxi Engineering Research Center of Eco-Remediation of Heavy Metal Pollution, Jiangxi Academy of Science Nanchang 330096 China

## Abstract

Phytoremediation is a potential cost-effective technology for remediating heavy metal-contaminated soils. This method was used to evaluate the biomass and accumulation of copper (Cu) and cadmium (Cd) of plant species grown in contaminated soil and their biological and physical effects on the soil. In co-contaminated soils with copper (Cu) and cadmium (Cd), a three-year field experiment was conducted by planting four plant species in the co-contaminated acidic soil treated with hydroxyapatite. The four species produced different amounts of biomass in this order: *Pennisetum* sp. > *Elsholtzia splendens* > *Setaria lutescens* > *Sedum plumbizincicola*. Over three growing seasons, the best accumulators of Cu and Cd were *Elsholtzia splendens* and *Sedum plumbizincicola*, respectively. Overall, *Pennisetum* sp. was the best species for Cu and Cd removal when biomass was considered. The four plant treatments could improve the content of >0.25 mm mechanically stable (DR_0.25_) and water-stable (WR_0.25_) aggregates and significantly improve the aggregate mean mass diameter (MWD) and the geometric mean diameter (GMD). The largest increase was with the treatment of *Pennisetum sinese*, while the fractal dimension (FD) of mechanically stable aggregates could be significantly reduced by the treatment of *Pennisetum* sp. Hydroxyapatite and phytoremediation could improve the soil enzyme activity, and *Elsholtzia splendens* had the best effect in this respect. This study will provide a better understanding of the remediation of heavy metal contaminated soil.

## Abstract 2

Phytoremediation is a potential cost-effective technology for remediating heavy metal-contaminated soils.

## Graphical abstract

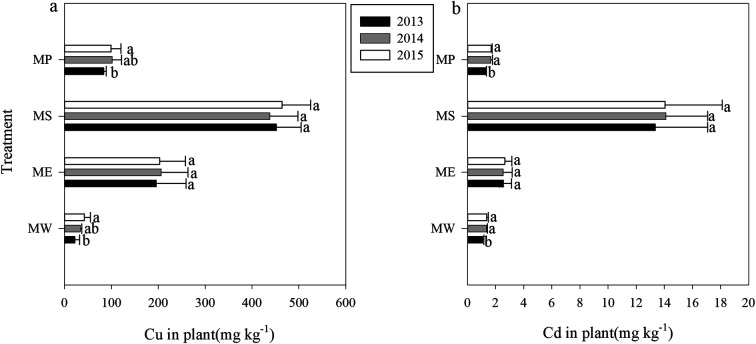

## 1. Introduction

Soil contamination, particularly, agricultural soil contamination, has become a severe environmental problem as it poses a grave threat to human health by entering food chains and to environmental safety by leaching into groundwater.^1^ Cadmium is a non-essential element that can cause great harm to plants and animals at very low concentrations. Excessive cadmium can not only cause serious pollution to soil, reducing crop yield and quality but can also be taken up by humans through the way of soil/crop/food migration, thus endangering human health.^2^ In fact, some pollutants are nowadays never isolated in the soil system. Cd can be brought into the soil when other heavy metal elements enter the soil. For example, in the process of copper smelting, not only copper but cadmium will also enter the soil together, thus leading to some contaminated soils.^3^

Various remediation methods, such as land filling, fixation, and leaching, may be beneficial to the remediation of Cu and Cd polluted soils.^4^ However, these methods are usually expensive, and some of them can impose adverse effects on the biological activity, structure, and fertility of soils.^5^ Phytoremediation is considered an environmentally friendly, gentle method of managing polluted sites as it uses biological processes to treat the pollutant.^6^ In contrast to most other remediation technologies, phytoremediation has significant environmental advantages.^7^ The application of native plants for phytoremediation is particularly important, because they can better adapt to the soil properties, toxicity levels and climatic conditions of the contaminated site.^8^ Gramineae species usually adapt faster to these conditions than other plant species because their shorter life cycles allow them to produce various genotypes in a shorter time.^9^ Some experiments have been carried out to investigate the phytoremediation potential of various plants in greenhouse experiments. At the same time, hyperaccumulators are often used, which can grow normally in soils contaminated with heavy metals and accumulate these metals in the harvested parts over the course of phytoremediation.^10^ In fact, in addition to hyperaccumulators, some plants with large biomass, good adaptability and fast growth rates also show good remediation potential for heavy metals, such as *Giantreed* and *Pennisetum* sp.^11^ However, in the process of remediation, because of the low soil pH, high metal toxicity may impede the growth of plants.^12^ Results have shown that some materials have good adsorption and stability to Cu and Cd such as ligno-cellulosic materials, *Saccharomyces cerevisiae*, biosorbents, limestone and hydroxyapatite.^13–15^ For example, the addition of hydroxyapatite can lead to an increase of soil pH; at the same time, it can reduce the toxicity of heavy metals through ion exchange reactions, surface complexation reactions, co-precipitation and precipitation.^10,16^ All these processes will promote the growth of plants in polluted areas. For example, Xu *et al.* found that *Pennisetum* sp. could grow normally only when lime was applied in the copper and cadmium contaminated soils.^17^

The aim of phytoremediation should not only be to remove heavy metals but also to improve soil quality.^18^ Therefore, the comprehensive evaluation of soil quality needs to be considered when evaluating the phytoremediation effect. Soil enzyme activity is the most active constituent; a large number of studies have shown that soil enzyme activity is sensitive to heavy metal pollution because of its significance in nutrient cycles, organic matter turnover, soil characteristics, microbial activity and biomass.^19,20^ The most frequently measured activities include b-1,4-glucosidase (BG, which catalyzes the terminal reaction in cellulose degradation), b-1,4-*N*-acetylglucosaminidase (NAG, which catalyzes the terminal reaction in chitin degradation), and acid or alkaline phosphatase (AP, hydrolyze phosphate esters including phosphomonoesters, phosphodiesters and, in some cases, phosphosaccharides that release phosphate), which are frequently linked to the rates of microbial metabolism and biogeochemical processes.^21^ At the same time, as a basic unit of soil structure, aggregate stability and its influencing factors are important for maintaining good soil structure and soil fertility.^22^ During phytoremediation, plants increase soil organic matter content through the decomposition of root exudates and litter, thus promoting the formation and stability of aggregates, improving soil physical and chemical properties.^23^ Therefore, it is necessary to evaluate the composition and structure of soil aggregates which can reflect the changes in soil physical properties during phytoremediation.

The goal of this study was to evaluate the metal removal efficiency of *Pennisetum* sp. compared with three other kinds of plants in combination with hydroxyapatite application in Cu and Cd contaminated soil. The objectives of this study include: (1) evaluating changes in soil chemical properties and heavy metal availability before and after the combined remediation; (2) determining removal efficiency and removal amount of heavy metals by different plants; (3) measuring the composition and stability of soil aggregates; and (4) measuring the soil enzyme activity.

## 2. Materials and methods

### 2.1. Study site

The study site is located in Guixi City, Jiangxi Province, China (117°12′E, 28°19′N). The area is located near a large copper smelter and fertilizer plant. There are about 130 hectares of soil in the area that are contaminated by heavy metals (mainly Cu and Cd) due to 30 years of sewage irrigation.^24^ At present, most of the soil in the area has been abandoned due to severe pollution, and desertification has appeared in some areas. The soil texture in this area is of sandy loam; the primary pollutants in the soil are Cu and Cd, with concentrations of 632 and 0.41 mg kg^−1^, respectively. Moreover, the site soil is very acidic (pH = 4.35), having soil organic carbon (SOC) content and cation exchange capacity (CEC) of 28.5 mg kg^−1^ and 8.31 cmol kg^−1^, respectively. Total N and total P in the soil were 1.11 g kg^−1^ and 0.190 g kg^−1^, respectively.

### 2.2. Reagent and plants

Hydroxyapatite (particle size = 0.25 mm, pH = 8.40, Cu and Cd concentrations were 9.54 mg kg^−1^ and 1.18 mg kg^−1^) was purchased from the Nanzhang Lihua mineral powder factory (Hubei, China). In this experiment, three species of plants were selected based on the high concentrations of Cu and Cd in the soil. We chose a copper tolerant plant (*Elsholtzia splendens*), a cadmium hyperaccumulator plant (*Sedum plumbizincicola*), and a kind of energy plant (*Pennisetum* sp.) that had a good tolerance to Cu and Cd in our previous study. All of the plants were derived from indoor-grown seedlings.

### 2.3. Experimental design

The field experiment consisted of 5 m (long) × 4 m (wide) plots arranged in a completely random plot design with three replicates per treatment. The treatments had four plants: native plant *Setaria lutescens* (MW), *Elsholtzia splendens* (ME), *Sedum plumbizincicola* (MS), and *Pennisetum* sp. (MP). A control treatment without plants or added hydroxyapatite (CK) was conducted in parallel. Prior to planting, 1% hydroxyapatite (based on the 0–17 cm soil weight) was applied and fully mixed into the soil by plowing on 23 December 2012. After a week of equilibration, a compound fertilizer (N : P_2_O_5_ : K_2_O = 15 : 15 : 15, 834 kg h m^−2^) was applied to each plot. The indigenous plants – *Setaria lutescens* could grow normally after the application of hydroxyapatite. For the other three treatments (ME, MS, and MP), *E. splendens*, *S. plumbizincicola*, and *Pennisetum* sp. were planted on 26 April each year (2013, 2014, and 2015). Weeds (mainly *S. lutescens*) were cleared from all plots before planting every year, and no weeding was carried out thereafter. The planting density was 20 cm × 20 cm for *E. splendens* and *S. plumbizincicola* plants, and 50 cm × 50 cm for *Pennisetum* sp. plants. All plots were managed using the same field management.

### 2.4. Sample collection

*S. plumbizincicola* was harvested in mid-July every year, while the upper part of the other plants were cut out of the ground by a sickle in mid-December every year. All the plant samples were taken to the laboratory and washed with tap water and then rinsed with ultrapure water. They were then put into an oven at 80 °C and dried until the weight no longer changed. Then, the sample was smashed with a grinder and put into a plastic bag. Soil samples were collected from each plot from an area of 20 cm × 20 cm × 17 cm, with three samples taken from each plot and then mixed together to form a mixed sample. These samples were air dried and sieved using a 5 mm sieve, and the resulting samples were used for the analysis of soil aggregates. Soil samples were collected from the top 17 cm at five representative locations per plot and then mixed together to form a composited sample after the plant harvest. The soil samples were divided into two parts: one part was dried and sifted for the analysis of soil physical and chemical properties, and the other part was kept at −80 °C for soil enzyme activity analysis.

### 2.5. Soil physicochemical and heavy metal analysis

Soil pH was determined based on the method of Xu and was measured using a pH meter (PHS-3CW-CN, Bante instrument, Shanghai, China).^10^ Soil organic carbon (SOC) was determined by the Walkley–Black procedure.^25^ Soil available nitrogen (N) and phosphate (P) were determined in the same way as Bingham, and soil available potassium (K) was measured in accordance with Olsen.^26,27^

Total soil Cu and Cd were measured in accordance with the method used by Cui, and a standard soil sample (GBW07405, National Research Center for Certified Reference Materials, China) was used to ensure the reliability of the experimental data.^28^ The available Cu and Cd in soils were extracted with 0.01 mol L^−1^ CaCl_2_ and determined in accordance with the method of Cui.^19^

### 2.6. Aggregates analysis

#### Mechanically stable aggregates

The mechanically stable aggregates was measured by the dry sieve method.^29^ The air-dried soil was put on the sieve with apertures of 5 mm, 2 mm, 1 mm, 0.5 mm and 0.25 mm. Then the aggregates were divided into six grades: >5 mm, 2–5 mm, 1–2 mm, 0.5–1 mm, 0.25–0.5 mm, and <0.25 mm. Then the proportion of each grade aggregate to soil weight was calculated based on the results.

#### Water stable aggregates

The water stable aggregates were measured by the method of Bearden.^30^ 100 g of soil samples were prepared according to the dry sieve ratio, placed in a 5 mm soil sieve and soaked in distilled water for 10 min. The soil samples were then passed through 2 mm, 1 mm, 0.5 mm, and 0.25 mm soil sieves, respectively. The aggregates were separated by moving the sieve 3 cm upward and downward 50 times (2 min); and then, the soil particles on the sieves were rinsed into the aluminum box, dried at 50 °C and weighed.

The contents of >0.25 mm mechanically stable aggregates (DR_0.25_) and >0.25 mm water-stable aggregates (WR_0.25_) were calculated based on the results. The mean weight diameter (MWD, mm), geometric mean diameter (GMD, mm) and fractal dimension (FD) of aggregates were calculated by Zhao's method.^31,32^1
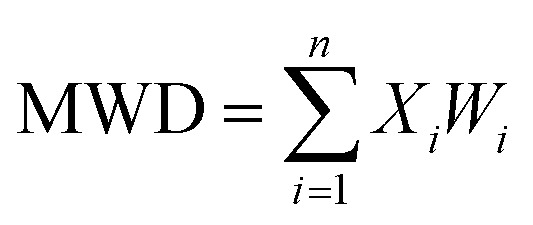
2
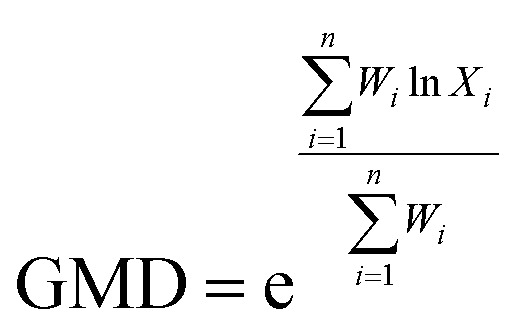
3
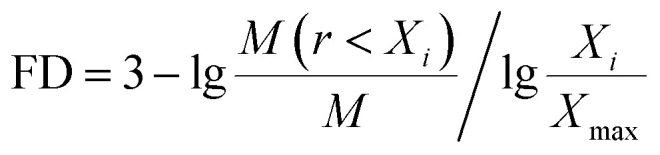


*X*_*i*_ is the average soil aggregate diameter at any level, equal to the average value of the adjacent two sieve hole stages. The upper limit of the diameter of >5 mm aggregates is 10 mm. *X*_max_ is the average particle size of the maximum particle size, mm, *M*(*r* < *X*_*i*_) is the weight of aggregates smaller than *X*_*i*_, and *M* is the aggregate weight. With 
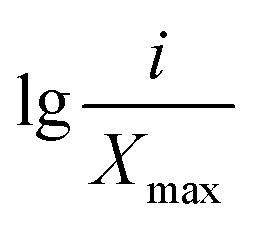
 as abscissa and 
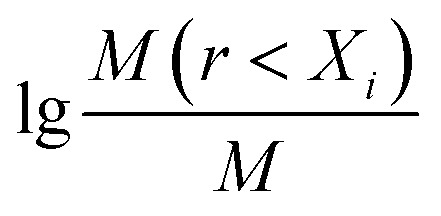
 as ordinate, the slope is calculated by linear fitting with the least squares method. Finally, the fractal dimension (FD) of mass is calculated from the slope.

### 2.7. Soil enzyme activity

The activities of b-glucosidase (BG), *N*-acetylglucosaminidase (NAG), and acid phosphatase (AP) were measured by the method of Saiya-Cork.^33^ Soils were assayed at ambient pH by suspending approximately 1 g of soil in 100 mL of 50 mm sodium acetate buffer. The microplates were incubated in the dark at 20 °C for 4 h. During the incubation, the incubation plates were shaken every hour to ensure the homogeneity of the reaction mixtures. To stop the reaction, a 1 mL aliquot of 1 M NaOH was added to each well.

### 2.8. Statistical analysis

All the data were presented as mean ± standard error and were estimated using one-way ANOVA at a significance level of 0.05 using SPSS 20.0 (IBM SPSS, Somers, NY, USA) when necessary. All the graphics in this article were made with Sigmaplot 12.5.

## 3. Results and discussion

### 3.1. Soil chemical characteristics and heavy metal availability

In the untreated soil (CK), the soil pH decreased from 4.24 to 4.20 during the three years (Table 1). The soil pH increased significantly from 4.24 to 5.17 when hydroxyapatite was added. This might be due to the high pH (8.40) of hydroxyapatite. This was consistent with Cui *et al.*, who found that the soil pH could be improved from of the addition of hydroxyapatite.^28^ At the same time, we found that plant growth did not significantly affect the soil pH (Table 1), although the plants might have secreted some weak organic acid ions, amino acids, vitamins, and inorganic ions (HCO_3_^−^, OH^−^, and H^+^) by the roots, which could change the soil pH.^34^ In the same way as the CK treatment, we found that the soil pH treated with hydroxyapatite also decreased slightly over time. This may be due to the fact that our experimental area was located in a typical acid rain area and that H^+^ in the atmosphere entered the soil, thus the reducing of the soil pH. The SOC concentration of the combination treatments (ME, MS, MP) increased significantly compared with CK after three years of remediation (Table 1). However, applying hydroxyapatite alone (MW) had no significant effect on SOC. This might be due to how the growing of plants could increase the amount of litter and fine roots and change the structure of soil aggregates, leading to an increase in SOC content.^35^ The phosphorus content in each treatment was significantly higher than that in the untreated soil because of the addition of hydroxyapatite. In southern China, phosphorus is generally deficient in soil, and the addition of hydroxyapatite is beneficial to mitigate the adverse effects of phosphorus deficiency in plants.

**Table:** Soil chemical characteristics after the harvest of four plant species during the three years. CK = untreated soil, MW = hydroxyapatite + *Setaria lutescens*, ME = hydroxyapatite + *Elsholtzia splendens*, MS = hydroxyapatite + *Sedum plumbizincicola*, MP = hydroxyapatite + *Pennisetum* sp. SOC, soil organic carbon; T-N, total nitrogen; T-P total phosphorus; CEC, cation exchange capacity; T-Cu, total Cu concentration; T-Cd, total Cd concentration. Different lowercase letters indicate significant differences between treatments at the same time (*n* = 3, *P* < 0.05)

Time	Treatment	pH	SOC g kg^−1^	T-N g kg^−1^	T-P g kg^−1^	CEC cmol kg^−1^	T-Cu mg kg^−1^	T-Cd mg kg^−1^	CaCl_2_–Cu mg kg^−1^	CaCl_2_–Cd mg kg^−1^
2013	CK	4.24 ± 0.207b	16.2 ± 0.191a	1.11 ± 0.0379a	0.190 ± 0.0100b	8.32 ± 0.0153a	666 ± 16.3a	0.412 ± 0.0244a	81.6 ± 25.9a	0.125 ± 0.0185a
	MW	5.17 ± 0.118a	16.3 ± 0.0458a	1.36 ± 0.0666a	0.620 ± 0.0872a	8.41 ± 0.149a	660 ± 28.1a	0.400 ± 0.0181a	28.4 ± 8.88b	0.081 ± 0.0123b
	ME	5.33 ± 0.0503a	17.8 ± 0.398a	1.32 ± 0.0900a	0.650 ± 0.0306a	8.63 ± 1.15a	618 ± 13.4a	0.390 ± 0.00913a	23.2 ± 5.44b	0.076 ± 0.0133b
	MS	5.19 ± 0.102a	18.0 ± 0.508a	1.26 ± 0.104a	0.780 ± 0.0723a	7.84 ± 0.974a	633 ± 19.3a	0.377 ± 0.0153a	22.8 ± 4.28b	0.066 ± 0.0162b
	MP	5.15 ± 0.135a	18.0 ± 1.39a	1.27 ± 0.173a	0.710 ± 0.202a	8.65 ± 0.575a	657 ± 15.3a	0.380 ± 0.0169a	23.4 ± 5.11b	0.073 ± 0.00932b
2014	CK	4.23 ± 0.110b	16.1 ± 0.172b	1.05 ± 0.0306a	0.190 ± 0.0379b	8.39 ± 0.0404a	674 ± 12.5a	0.394 ± 0.0142a	94.2 ± 36.3a	0.132 ± 0.0283a
	MW	5.16 ± 0.130a	16.2 ± 0.118b	1.40 ± 0.0305a	0.620 ± 0.0764a	8.53 ± 0.285a	664 ± 23.7a	0.391 ± 0.0155a	31.1 ± 6.95b	0.086 ± 0.00768ab
	ME	5.24 ± 0.261a	18.4 ± 0.725a	1.33 ± 0.112a	0.520 ± 0.0404a	8.64 ± 0.170a	621 ± 16.9a	0.372 ± 0.00811a	34.5 ± 13.2b	0.073 ± 1.73 × 10^−2^b
	MS	5.13 ± 0.195a	18.4 ± 0.239a	1.25 ± 0.123a	0.680 ± 0.0608a	8.47 ± 0.180a	639 ± 29.0a	0.359 ± 0.0256a	26.0 ± 13.5b	0.068 ± 0.0215b
	MP	5.14 ± 0.253a	18.8 ± 0.546a	1.27 ± 0.251a	0.630 ± 0.242a	8.56 ± 0.206a	650 ± 25.2a	0.352 ± 0.0346a	25.0 ± 3.30b	0.075 ± 0.0113b
2015	CK	4.20 ± 0.280b	16.5 ± 0.451b	1.08 ± 0.0600b	0.190 ± 0.0153b	8.36 ± 0.140b	668 ± 11.7a	0.406 ± 0.0154a	100.0 ± 21.1a	0.148 ± 0.0175a
	MW	5.03 ± 0.137a	16.6 ± 0.358b	1.45 ± 0.0379a	0.510 ± 0.136a	8.51 ± 0.0929b	658 ± 24.5a	0.389 ± 0.0124ab	49.8 ± 7.48b	0.119 ± 0.0123ab
	ME	5.26 ± 0.172a	19.0 ± 0.593a	1.46 ± 0.0436a	0.460 ± 0.0252ab	8.84 ± 0.0710a	616 ± 8.67ab	0.364 ± 0.00526b	45.9 ± 6.66b	0.118 ± 0.00747ab
	MS	5.13 ± 0.161a	18.6 ± 1.09a	1.28 ± 0.0751ab	0.620 ± 0.102a	8.53 ± 0.0200b	630 ± 8.53ab	0.350 ± 0.0120b	43.5 ± 3.50b	0.090 ± 0.0211b
	MP	5.07 ± 0.0971a	19.5 ± 0.478a	1.35 ± 0.276ab	0.550 ± 0.140a	8.56 ± 0.0819b	649 ± 13.8b	0.350 ± 0.0227b	44.3 ± 9.81b	0.089 ± 0.0279b

It is known that the harmfulness of heavy metals in soils is mainly determined by their availability and mobility, CaCl_2_-extractable heavy metals can be used as an index to measure the availability of heavy metals in soil.^36^ Our experimental results indicated that untreated soil had the highest CaCl_2_ extractability (Cu 81.6 mg kg^−1^, Cd 0.125 mg kg^−1^ in 2013). The addition of hydroxyapatite significantly reduced the available Cu and Cd in the soil; the lowest extractable Cu by CaCl_2_ (Cu 43.5 mg kg^−1^) was in *Sedum plumbizincicola* plots and Cd by CaCl_2_ (Cd 0.089 mg kg^−1^) was in *Pennisetum* sp. plots (Table 1). The results showed that potential mobility of Cu and Cd in the control was higher than that in hydroxyapatite treated soils, which might be mainly due to the lower pH in the CK.

### 3.2. Biomass and metal accumulation

**Fig. 1:**
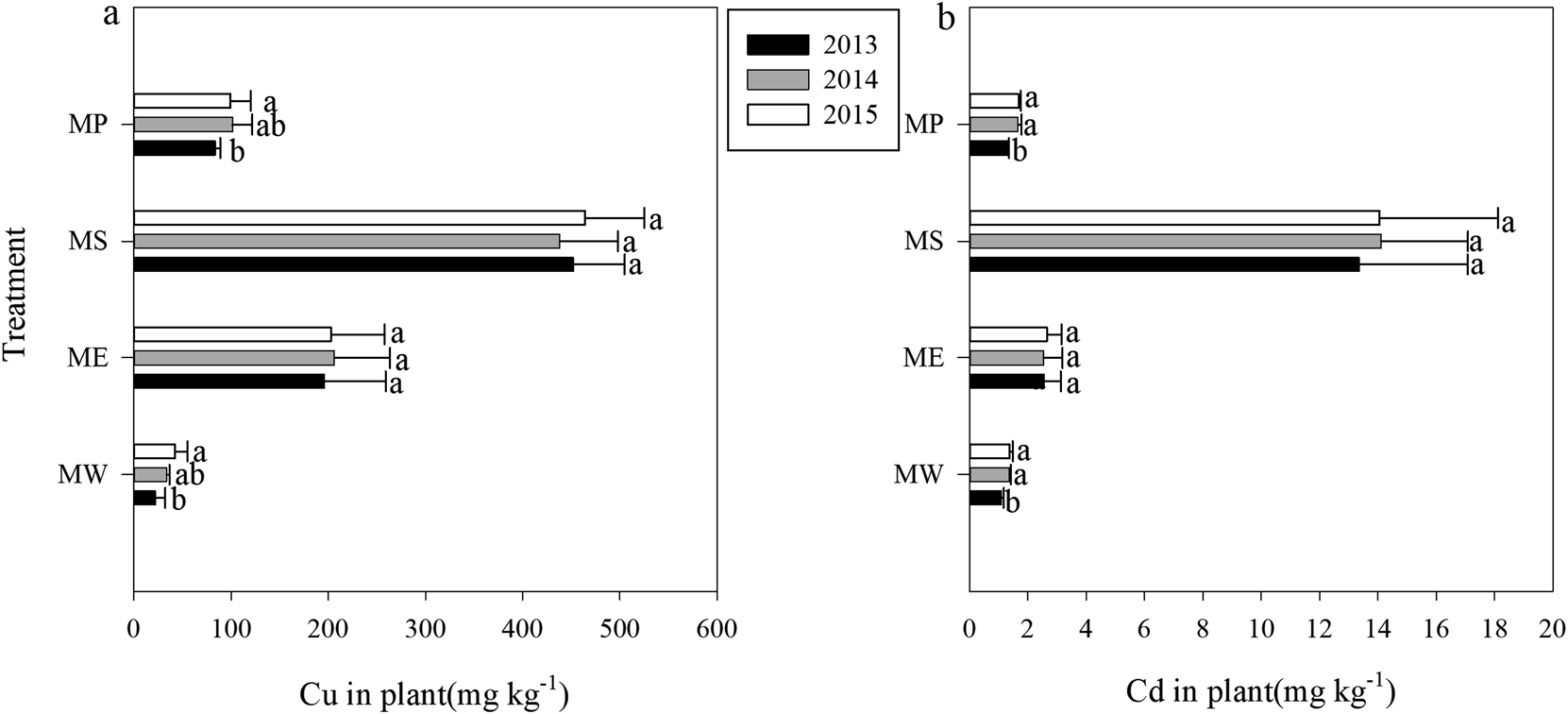
Concentrations of (a) Cu and (b) Cd in the shoots of each plant. MW = *Setaria lutescens*, ME = *Elsholtzia splendens*, MS = *Sedum plumbizincicola*, MP = *Pennisetum* sp. Different lowercase letters indicate significant differences in the same treatment during the three years (*n* = 3, *P* < 0.05).

In our study, the native *S. lutescens* and the three phytoextractors were able to grow normally only after hydroxyapatite application. Among the four plants grown, the biomass of *Pennisetum* sp. was the largest followed by *Elsholtzia splendens*, *Setaria lutescens* and *Sedum plumbizincicola* (Table 2). As a hyperaccumulator of cadmium, *Sedum plumbizincicola* exhibited a high absorptive capacity for Cu (451.5 mg kg^−1^) and Cd (13.7 mg kg^−1^) in our study, which were 13.8 and 11.8 times that of *Setaria lutescens*, respectively. *E. splendens* (a Cutolerant plant) also showed a high absorption capacity for Cu and Cd, which reached 202 mg kg^−1^ and 2.59 mg kg^−1^, respectively (Table 2, Fig. 1).

**Table:** Shoot biomass and Cu and Cd accumulation in each plant during phytoextraction. CK = untreated soil, MW = hydroxyapatite + *Setaria lutescens*, ME = hydroxyapatite + *Elsholtzia splendens*, MS = hydroxyapatite + *Sedum plumbizincicola*, MP = hydroxyapatite + *Pennisetum* sp. Different lowercase letters indicate significant differences between treatments in the same year (*n* = 3, *P* < 0.05). — indicates no plant growth

Treatment	Shoot biomass (t dry weight h per m^2^ per year)			Metal accumulation (g h per m^2^ per year)					
				Cu				Cd	
	2013	2014	2015	2013	2014	2015	2013	2014	2015
CK	—	—	—	—	—	—	—	—	—
MW	10.1 ± 4.91bc	8.55 ± 1.52bc	5.20 ± 0.560c	236 ± 148c	285 ± 20.5b	224 ± 93.2b	10.4 ± 3.96b	10.3 ± 4.27b	6.50 ± 2.23c
ME	15.1 ± 4.17ab	12.6 ± 1.38b	14.4 ± 4.22b	2.74 × 10^3^ ± 437a	2.54 × 10^3^ ± 759a	2.93 × 10^3^ ± 1.28 × 10^3^a	39.2 ± 15.0a	32.1 ± 8.59a	37.6 ± 8.49b
MS	2.25 ± 0.365c	2.10 ± 0.210c	2.70 ± 0.468c	1.03 × 10^3^ ± 266c	910 ± 92.8b	1.28 × 10^3^ ± 395ab	29.8 ± 3.94ab	29.5 ± 1.10a	38.1 ± 5.15ab
MP	22.3 ± 3.36a	29.2 ± 6.10a	37.7 ± 4.14a	1.88 × 10^3^ ± 353b	2.98 × 10^3^ ± 949a	3.81 × 10^3^ ± 1.40 × 10^3^a	29.1 ± 4.46ab	39.8 ± 8.97a	52.0 ± 3.94a

According to Pedron, phytoremediation can be considered as a successful treatment for soil contaminated with heavy metals if plants are able to reduce soil metal concentration over time through the uptake processes.^37^ Thus, “removal efficiency” should be calculated using tissue concentration and biomass produced to illustrate the treatment effectiveness of phytoextraction.^38^ With regard to the total accumulation of Cu and Cd, *Pennisetum* sp. showed the greatest advantage, with the three-year cumulative amounts of 8.67 × 10^3^ g h m^−2^ and 121 g h m^−2^, respectively (Table 2). The integrated results of plant biomass and accumulation capability showed that *E. splendens* and *S. plumbizincicola* had similar absolute accumulations of Cu and Cd. The poor biomass and accumulation ability of *S. lutescens* showed that this native plant species had the lowest absolute accumulation. In terms of absolute accumulation concentration, the remediation efficiency of different plants ranked *Pennisetum* sp. > *E. splendens* > *S. plumbizincicola* > *S. lutescens*. Based on our data, we would suggest that intercropping *S. plumbizincicola* with *Pennisetum* sp. or *E. splendens* might be a useful approach to removing Cu and Cd from soils. Nevertheless, more experiments are still needed to verify this hypothesis.

### 3.3. Soil aggregates structure and stability

As a basic component of soil, aggregates play an important role in the transportation of water, nutrients and air in soil. The improvement of soil aggregate stability is conducive to the progress of these processes.^39^ In the process of evaluating the stability of soil aggregates, the average mass diameter, geometric mean diameter and aggregate stability rate are commonly used indicators. Using these indicators, we can objectively evaluate the stability of soil aggregates.^40^ The content of the soil with mechanically stable aggregates was 69.4–76.6% before the harvest of vegetation in 2015 (Table 3), which was less than a lot of previous studies.^41,42^ This indicated that the physical structure of the soil in this area was poor, which might be related to serious soil pollution and difficult growth of plants in the area, resulting in the slight desertification of soil and the deterioration of soil structure.^43^

Effects of phytoremediation on the composition of soil aggregates. CK = untreated soil, MW = hydroxyapatite + *Setaria lutescens*, ME = hydroxyapatite + *Elsholtzia splendens*, MS = hydroxyapatite + *Sedum plumbizincicola*, MP = hydroxyapatite + *Pennisetum* sp. Different lowercase letters indicate significant differences between treatments in the same year (*n* = 3, *P* < 0.05)

**Table:** 

Treatment	The size of soil mechanically stable aggregates						
	>5 mm	5–2 mm	2–1 mm	1–0.5 mm	0.5–0.25 mm	<0.25 mm	DR_0.25_
CK	10.2 ± 0.405b	26.0 ± 1.19b	8.34 ± 0.95a	11.0 ± 1.10b	13.9 ± 0.493a	30.2 ± 1.46a	69.4 ± 1.34b
MW	11.2 ± 0.674ab	27.1 ± 0.322ab	9.23 ± 0.277a	13.7 ± 1.23a	12.0 ± 0.607b	25.7 ± 1.33b	73.2 ± 1.09ab
ME	10.4 ± 0.356b	27.6 ± 1.21ab	9.35 ± 0.815a	14.1 ± 0.813a	12.5 ± 0.569b	25.3 ± 2.35b	74.0 ± 2.64a
MS	11.5 ± 0.654ab	28.0 ± 1.05ab	8.83 ± 0.464a	14.1 ± 0.201a	12.3 ± 0.222b	24.6 ± 0.933b	74.6 ± 0.90a
MP	13.1 ± 1.30a	28.6 ± 0.167a	8.96 ± 1.27a	13.2 ± 0.745ab	12.6 ± 0.628ab	22.8 ± 1.00b	76.6 ± 0.73a

**Table:** 

Treatment	The size of soil water-stable aggregates						
	>5 mm	5–2 mm	2–1 mm	1–0.5 mm	0.5–0.25 mm	<0.25 mm	WR_0.25_
CK	5.18 ± 0.630b	7.26 ± 1.60a	6.00 ± 0.265b	8.29 ± 0.66ab	18.0 ± 3.39a	55.3 ± 2.97a	44.7 ± 2.97b
MW	5.25 ± 0.606b	8.95 ± 0.514a	6.43 ± 0.594b	8.21 ± 0.374ab	19.8 ± 0.974ab	50.7 ± 1.35ab	48.7 ± 1.33ab
ME	6.41 ± 0.704ab	8.83 ± 1.55a	6.68 ± 0.182b	7.59 ± 1.13b	22.8 ± 1.39a	47.7 ± 2.32b	52.3 ± 2.32a
MS	5.32 ± 0.768b	7.98 ± 1.33a	6.70 ± 0.101b	7.00 ± 0.248b	23.7 ± 2.33a	49.3 ± 2.27b	50.7 ± 2.27a
MP	7.70 ± 0.887a	7.49 ± 0.599a	8.39 ± 0.445a	9.64 ± 0.130a	19.3 ± 0.610a	47.4 ± 1.16b	52.6 ± 1.16a

The content of DR_0.25_ in soil significantly increased after 3 years of remediation for all three kinds of plants; moreover, the 3 plant treatments mainly increased the mechanically stable aggregate content of >5 mm, 2–5 mm and 0.5–1 mm, while especially increasing the content of >2 mm aggregates. This showed that the remediation of the three plants had a significant promoting effect on the formation of soil with >0.25 mm mechanically stable aggregates, which was mainly achieved by increasing the content of the >2 mm mechanically stable aggregates. Among the 3 plants, the maximum increase of >0.25 mm aggregates was *Pennisetum* sp., especially for the >5 mm and 2–5 mm aggregates, which increased by 29.0% and 10.1% compared with the CK treatment, respectively. The results showed that *Pennisetum* sp. had the largest increase in mechanically stable aggregates and had the best effect on improving soil physical structure. The percent content of water-stable aggregates for different treatments were 44.7–52.6% (Table 3). Similar to mechanically stable aggregates, all three kinds of plants could significantly increase the content of water-stable aggregates. The increase was mainly concentrated in the >5 mm aggregates, and it was not significant in other particle sizes. This was the same as that of Zheng, who found that the vegetation restoration process mainly increased the content of >5 mm aggregates.^44^

The mean weight diameter (MWD) and geometric mean diameter (GMD) are important indexes for evaluating the stability of soil aggregates. The increase of MWD and GMD values can represent the increase in soil aggregate stability.^45^ The MWD and GMD of mechanically stable and water-stable aggregates increased significantly after the 3 years of remediation in our study (Table 4). The MWD and GMD values of both mechanically stable and water-stable aggregates increased to the greatest extent. In terms of MWD, MP treatment increased the amount of mechanically stable and water-stable aggregates by 16.2% and 24.2% compared with CK treatment. In terms of GMD, the GMD of mechanically stable and water-stable aggregates, compared to CK, increased 29.1% and 25.0% by MP treatment, respectively. The results proved again that the application of hydroxyapatite and *Pennisetum* sp. in the phytoremediation of degraded heavy metal contaminated soil could improve the stability of soil aggregates and the physical structure of the soil. Moreover, the MWD and GMD of the mechanically stable aggregate were greater than those of water-stable aggregates in all the treatments, which indicated that the mechanically stable aggregates were the main aggregate type in the soil. These results were consistent with those of Zhang *et al.*^46^

**Table:** Effects of vegetation restoration on the mean weight diameter and geometric mean diameter of mechanically stable and water-stable aggregates in heavy metal contaminated soil. CK = untreated soil, MW = hydroxyapatite + *Setaria lutescens*, ME = hydroxyapatite + *Elsholtzia splendens*, MS = hydroxyapatite + *Sedum plumbizincicola*, MP = hydroxyapatite + *Pennisetum* sp. Different lowercase letters indicate significant differences between treatments in the same year (*n* = 3, *P* < 0.05)

Treatment	Mean weight diameter (MWD) (mm)		Geometry weight diameter (GMD) (mm)		Fractal dimension (FD)	
	Mechanical-stable aggregates	Water-stable aggregates	Mechanical-stable aggregates	Water-stable soil aggregates	Mechanical-stable aggregates	Water-stable soil aggregates
CK	1.98 ± 0.0374c	0.946 ± 0.0944b	0.794 ± 0.0264c	0.324 ± 0.0205b	3.15 ± 0.0964a	3.54 ± 0.0563a
MW	2.11 ± 0.0382bc	1.02 ± 0.0593ab	0.915 ± 0.0303b	0.358 ± 0.0150ab	3.10 ± 0.0163b	3.53 ± 0.0431a
ME	2.08 ± 0.0614bc	1.10 ± 0.0733ab	0.909 ± 0.0532b	0.379 ± 0.0220a	3.09 ± 0.0205b	3.47 ± 0.0357a
MS	2.16 ± 0.0350b	1.00 ± 0.0917ab	0.947 ± 0.0237ab	0.353 ± 0.0216ab	3.07 ± 0.0139b	3.52 ± 0.0480a
MP	2.30 ± 0.0731a	1.18 ± 0.0648a	1.02 ± 0.0287a	0.398 ± 0.170a	3.03 ± 0.0148c	3.44 ± 0.0260a

Fractal dimension (FD) is a new index to used evaluate the comprehensive soil structure. It can reflect the stability of soil aggregates while reflecting the uniformity of soil texture. The lower the fractal dimension, the more beneficial to the improvement of soil nutrient circulation and structure.^47^ The study found that the fractal dimension (FD) of mechanically stable aggregates could be significantly reduced by the restoration of 4 planting. The range of the reduction range was 1.59–3.81% (Table 4). This showed that after 3 years of vegetation restoration, the particle size composition of the soil aggregates was more homogeneous and the physical structure of soil had been improved.

Soil organic carbon is an important index for evaluating soil quality, which has an important influence on the formation and cementation of aggregates. As an existing place for soil organic carbon, aggregates play an important role in the storage of organic matter and the transport of water vapor. Therefore, soil organic carbon and aggregates are inseparable.^48^ After 3 years of vegetation restoration on this heavy metal contaminated soil, the content of soil organic matter and >0.25 mm aggregates increased significantly, while the improvement of *Pennisetum* sp. treatment which had the highest biomass per unit area was the most significant. Regression analysis showed that soil organic matter content was positively correlated with >0.25 mm mechanically stable aggregates (DR_0.25_) and water-stable aggregates (WR_0.25_) (*R*^2^ = 0.550*, 0.504*). This result was in accordance with the results of Zhu *et al.*, who found that the main reason for the formation and increase of large aggregates was the increase in organic matter content.^49^ The restoration of vegetation improved the content of organic matter and organic residues in the soil, and the smaller aggregates in the soil formed a larger aggregate by cementing organic carbon, mycelium nuclei and plant residues in the soil.^50^

### 3.4. Soil enzyme activities

**Fig. 2:**
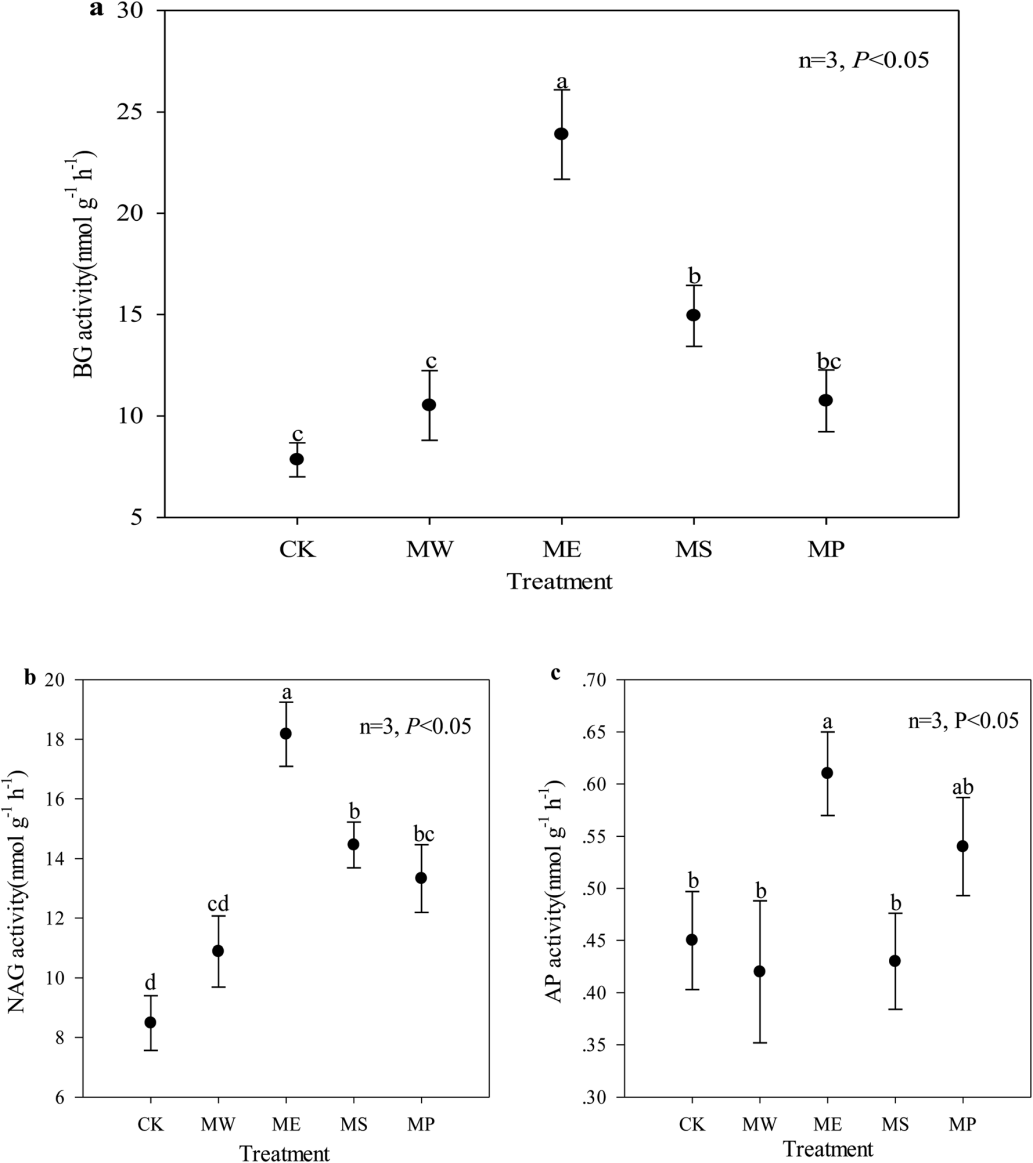
Soil enzyme activities after the harvest of the four plant species in 2015. (a) BG activity, (b) NAG activity, (c) AP activity. CK = untreated soil, MW = hydroxyapatite + *Setaria lutescens*, ME = hydroxyapatite + *Elsholtzia splendens*, MS = hydroxyapatite + *Sedum plumbizincicola*, MP = hydroxyapatite + *Pennisetum* sp. BG, b-1,4-glucosidase; NAG, b-1,4-*N*-acetylglucosaminidase; AP, acid phosphatase. Different lowercase letters indicate significant differences between treatments obtained at the same time.

Soil enzyme activity is a direct indicator of soil microbial activity in response to metabolic requirements and available nutrients; it is especially useful for evaluating the impact of heavy metal pollution in soil.^51^ The untreated soil showed very low BG and NAG activities, indicating a poor functional ability to catalyze the decomposition and transformation of soil carbon and nitrogen. The activities of BG, NAG and AP increased significantly by 205%, 114% and 17.4% in *E. splendens* soil, respectively, as compared with the control (Fig. 2). But the activities of these three enzymes were all at low levels in the *Setaria lutescens* plot. In contrast to BG and NAG, AP activity was only slightly affected by the treatments except for the *E. splendens* plots. This might be due to the fact that acid conditions were favorable to acid phosphatase activity.^52^ Previous studies have reported that heavy metals in soils can inhibit enzyme activities (1) by their toxic effects on soil microflora, (2) by combining with the active groups of the enzymes (3) through the complexation of the substrate, and (4) by reacting with the enzyme–substrate complex.^53,54^ O. N. Belyaeva *et al.* found that soil BG and NAG activities increased with decreasing soil bioavailable Cu.^55^

Interestingly, in addition to the significant correlation between NAG activity and CaCl_2_-extractable copper, there was no significant correlation among the other two enzyme activities and CaCl_2_-extractable copper and cadmium (Table 5). BG and NAG activity was mainly significantly positively correlated with soil pH and CEC but negatively correlated with total Cu in the soil. The soil pH and cation exchange affect the immobilization of enzymes in the soil.^56^ The potential for biological activity as indicated by the enzyme activity may be attributed to root residues and root exudates (such as amino acids, sugars, phenolics, polysaccharides and proteins). Such processes could improve soil physical and chemical properties and cause changes in the composition and function of soil microbial communities.^57^ In our study, soil pH was an important factor that restricted plant growth; the increase of soil pH could promote the activity of soil BG and NAG activity. AP contributes to the transformation of organic phosphorus to inorganic phosphorus, thereby enhancing the absorption of inorganic phosphorus by plants.^58^ The synthesis of new phosphatase or the phosphatase activity in the soil can be inhibited by inorganic phosphate.^59^ In this study, the addition of hydroxyapatite led to the increase of phosphate, which could lead to the decrease of AP activity. On the other hand, the addition of hydroxyapatite led to the increase of soil pH and to the reduced toxicity of heavy metals in the soil, which could improve the activity AP. The combined effect resulted in a small increase in the activity of AP (Fig. 2c).

**Table:** Correlation coefficients among soil biological properties, soil chemical properties, and CaCl_2_-extractable Cu and Cd. (BG, b-1,4-glucosidase; NAG, b-1,4-*N*-acetylglucosaminidase; AP, acid phosphatase; CEC, cation exchange capacity; T-Cu, total Cu; T-Cd, total Cd; C-Cu, CaCl_2_-extractable Cu; C-Cd, CaCl_2_-extractable Cd; SOC, soil organic carbon; T-N, soil total nitrogen; T-P soil total phosphorus. All samples of all plots included in these correlation analyses (*n* = 18). ** Correlation is significant at the 0.01 level; * correlation is significant at the 0.05 level)

	BG	NAG	AP	pH	CEC	T-Cu	T-Cd	C-Cu	C-Cd	SOC	T-N	T-P
BG	1.00											
NAG	0.861*	1.00										
AP	0.564*	0.600*	1.00									
pH	0.607*	0.793**	0.271	1.00								
CEC	0.848**	0.836**	0.537**	0.698**	1.00							
T-Cu	−0.783**	−0.797**	−0.296	−0.559*	−0.675**	1.00						
T-Cd	−0.382	−0.651**	−0.428	−0.618*	−0.401	0.470	1.00					
C-Cu	−0.474	−0.645**	−0.240	−0.733**	−0.464	0.599*	0.742**	1.00				
C-Cd	−0.180	−0.465	−0.135	−0.514*	−0.247	0.268	0.793**	0.757**	1.00			
SOC	0.505	0.647**	0.622*	0.523*	0.488	−0.515*	−0.702**	−0.573*	−0.489	1.00		
T-N	0.487	0.446	0.250	0.677**	0.506	−0.310	−0.120	−0.525*	−0.0396	0.371	1.00	
T-P	0.272	0.465	−0.0142	0.689**	0.235	−0.460	−0.565*	−0.779**	−0.520*	0.518*	0.609*	1.00

## 4. Conclusions

This study demonstrates the benefits of combining hydroxyapatite application and phytoextraction for the improvement of soil quality when remediating heavy metals. Four plant species were successfully established in the hydroxyapatite-amended soil and produced different amounts of aboveground biomass in the order of *Pennisetum* sp. > *Elsholtzia splendens* > *Setaria lutescens* > *Sedum plumbizincicola*. Results indicated that *Pennisetum* sp. was the best species for Cu and Cd removal from the contaminated soils. The application of hydroxyapatite and four plant treatments could improve the content of >0.25 mm mechanically stable (DR_0.25_) and water-stable (WR_0.25_) aggregates and the stability of soil aggregates, the largest increase being with the treatment of *Pennisetum sinese*. In addition, hydroxyapatite and phytoremediation could improve soil enzyme activity, and *Elsholtzia splendens* had the best effect in this respect. In conclusion, *Elsholtzia splendens* and *Pennisetum* sp. may be the best choices for the remediation of this type of heavy metal contaminated soil.

## Conflicts of interest

There are no conflicts to declare.

## Supplementary Material

## References

[cit1] Hechmi N., Ben A. N., Abdennaceur H., Jedidi N. (2013). Phytoremediation potential of maize (*Zea mays* L.) in co-contaminated soils with pentachlorophenol and cadmium. Int. J. Phytorem..

[cit2] Yin X., Xu Y., Huang R., Huang Q., Xie Z., Cai Y., Liang X. (2017). Remediation mechanisms for Cd-contaminated soil using natural sepiolite at the field scale. Environ. Sci.: Processes Impacts.

[cit3] Gautam S. K., Maharana C., Sharma D., Singh A. K., Tripathi J. K., Singh S. K. (2015). Evaluation of groundwater quality in the Chotanagpur plateau region of the Subarnarekha river basin, Jharkhand State, India. Sustainability of Water Quality and Ecology.

[cit4] Lu K., Yang X., Gielen G., Bolan N., Ok Y. S., Niazi N. K., Xu S., Yuan G., Chen X., Zhang X. (2017). Effect of bamboo and rice straw biochars on the mobility and redistribution of heavy metals (Cd, Cu, Pb and Zn) in contaminated soil. J. Environ. Manage..

[cit5] Ali H., Khan E., Sajad M. A. (2013). Phytoremediation of heavy metals--concepts and applications. Chemosphere.

[cit6] Greipsson S. (2011). Phytoremediation. Nature Education Knowledge.

[cit7] Vithanage M., Dabrowska B. B., Mukherjee A. B., Sandhi A., Bhattacharya P. (2012). Arsenic uptake by plants and possible phytoremediation applications: a brief overview. Environ. Chem. Lett..

[cit8] Pilonsmits E. (2005). Phytoremediation. Annu. Rev. Plant Biol..

[cit9] Conesa H. M., Moradi A. B., Robinson B. H., Kühne G., Lehmann E., Schulin R. (2009). Response of native grasses and *Cicer arietinum* to soil polluted with mining wastes: implications for the management of land adjacent to mine sites. Environ. Exp. Bot..

[cit10] Xu L., Cui H., Zheng X., Zhu Z., Liang J., Zhou J. (2016). Immobilization of copper and cadmium by hydroxyapatite combined with phytoextraction and changes in microbial community structure in a smelter-impacted soil. RSC Adv..

[cit11] Marchiol L., Assolari S., Sacco P., Zerbi G. (2004). Phytoextraction of heavy metals by canola (*Brassica napus*) and radish (*Raphanus sativus*) grown on multicontaminated soil. Environ. Pollut..

[cit12] Pardo T., Clemente R., Bernal M. P. (2011). Effects of compost, pig slurry and lime on trace element solubility and toxicity in two soils differently affected by mining activities. Chemosphere.

[cit13] Huang Y., Yang C., Sun Z., Zeng Z., He H. (2015). Removal of cadmium and lead from aqueous solutions using nitrilotriacetic acid anhydride modified ligno-cellulosic material. RSC Adv..

[cit14] Wang J., Chen C. (2006). Biosorption of heavy metals by *Saccharomyces cerevisiae*: a review. Biotechnol. Adv..

[cit15] Wang J., Chen C. (2009). Biosorbents for heavy metals removal and their future. Biotechnol. Adv..

[cit16] Karami N., Clemente R., Morenojiménez E., Lepp N. W., Beesley L. (2011). Efficiency of green waste compost and biochar soil amendments for reducing lead and copper mobility and uptake to ryegrass. J. Hazard. Mater..

[cit17] Xu L., Zhou J., Liang J., Cui H., Tao M., Tao Z., Zhu Z., Huang L. (2014). The remediation potential of *Pennisetum* sp. On Cu, Cd contaminated soil. Acta Ecol. Sin..

[cit18] Jusselme M. D., Miambi E., Mora P., Diouf M., Rouland-Lefèvre C. (2013). Increased lead availability and enzyme activities in root-adhering soil of *Lantana camara* during phytoextraction in the presence of earthworms. Sci. Total Environ..

[cit19] Cui H. B., Fan Y. C., Zhou J., Shi Y., Xu L., Guo X. T., Hu Y. B., Gao L. M. (2016). Availability of soil Cu and Cd and microbial community structure as affected by applications of amendments. China Environ. Sci..

[cit20] Pérez-De-Mora A., Burgos P., Madejón E., Cabrera F., Jaeckel P., Schloter M. (2006). Microbial community structure and function in a soil contaminated by heavy metals: effects of plant growth and different amendments. Soil Biol. Biochem..

[cit21] Shahzad S. M., Khalid A., Arif M. S., Riaz M., Ashraf M., Iqbal Z. (2014). Co-inoculation integrated with P-enriched compost improved nodulation and growth of Chickpea (*Cicer arietinum* L.) under irrigated and rainfed farming systems. Biol. Fertil. Soils.

[cit22] Yoshinobu S., Tomo'Omi K., Atsushi K., Kyoichi O., Shigeru O. (2004). Experimental analysis of moisture dynamics of litter layers – the effects of rainfall conditions and leaf shapes. Hydrol. Processes.

[cit23] Wang Q. Y., Liu J. S., Wang Y., Yu H. W. (2015). Accumulations of copper in apple orchard soils: distribution and availability in soil aggregate fractions. J. Soils Sediments.

[cit24] Ping L. I., Wang X., Zhang T., Zhou D., Yuanqiu H. E. (2008). Effects of several amendments on rice growth and uptake of copper and cadmium from a contaminated soil. J. Environ. Sci..

[cit25] Tiwari M. K. N., Nigam V., Pathak A. N. (1982). Effect of potassium and zinc applications on dry-matter production and nutrient uptake by potato variety ‘Kufri chandramukhi’ (*Solanum tuberosum* L.) in an alluvial soil of Uttar Pradesh. Plant Soil.

[cit26] Sparks D. L., Page A. L., Helmke P. A., Loeppert R. H. (1996). Redox measurements of soils. Plast. Reconstr. Surg..

[cit27] Olsen S. R. , Estimation of available phosphorus in soils by extraction with sodium bicarbonate, Institute for Agricultural Research Samaru, 1954

[cit28] Cui H. B., Zhou J., Si Y. B., Mao J. D., Zhao Q. G., Fang G. D., Liang J. N. (2014). Immobilization of Cu and Cd in a contaminated soil: one- and four-year field effects. J. Soils Sediments.

[cit29] Zhang X., Chen L., Fu B., Li Q., Qi X., Ma Y. (2006). Soil organic carbon changes as influenced by agricultural land use and management: a case study in Yanhuai Basin, Beijing, China. Acta Ecol. Sin..

[cit30] Bearden B. N., Petersen L. (2000). Influence of arbuscular mycorrhizal fungi on soil structure and aggregate stability of a vertisol. Plant Soil.

[cit31] Fonte S. J., Yeboah E., Ofori P., Quansah G. W., Vanlauwe B., Six J. (2009). Fertilizer and residue quality effects on organic matter stabilization in soil aggregates. Soil Sci. Soc. Am. J..

[cit32] Zhao Y. S., Han C. H., Zhang H. G., Chen Q., Liu M., Han X. C. (2012). Soil Hydrologic Functions of Main Forest Types in Ashi River's Upstream Watershed. J. Soil Water Conserv..

[cit33] Saiya-Cork K. R., Sinsabaugh R. L., Zakb D. R. (2002). The effects of long term nitrogen deposition on extracellular enzyme activity in an *Acer saccharum* forest soil. Soil Biol. Biochem..

[cit34] Haichar F. E. Z., Santaella C., Heulin T., Achouak W. (2014). Root exudates mediated interactions belowground. Soil Biol. Biochem..

[cit35] Wu G. L., Liu Y., Tian F. P., Shi Z. H. (2017). Legumes Functional Group Promotes Soil Organic Carbon and Nitrogen Storage by Increasing Plant Diversity. Land Degradation & Development.

[cit36] Huang J. H., Hsu S. H., Wang S. L. (2011). Effects of rice straw ash amendment on Cu solubility and distribution in flooded rice paddy soils. J. Hazard. Mater..

[cit37] Pedron F., Petruzzelli G., Barbafieri M., Tassi E. (2009). Strategies to use phytoextraction in very acidic soil contaminated by heavy metals. Chemosphere.

[cit38] Jarrell W. M., Beverly R. B. (1981). The dilution effect in plant nutrition studies. Adv. Agron..

[cit39] Li W., Zheng Z., Li T., Zhang X., Wang Y., Yu H., He S., Liu T. (2015). Effect of tea plantation age on the distribution of soil organic carbon fractions within water-stable aggregates in the hilly region of Western Sichuan, China. Catena.

[cit40] Sieling K., Herrmann A., Wienforth B., Taube F., Ohl S., Hartung E., Kage H. (2013). Biogas cropping systems: short term response of yield performance and N use efficiency to biogas residue application. Eur. J. Agron..

[cit41] Jie L. I., Yang X. Y., Sun B. H., Zhang S. L., Aamp N. (2014). Effects of soil management practices on stability and distribution of aggregates in Lou soil. Journal of Plant Nutrition & Fertilizer.

[cit42] Liu Z., Chen X., Jing Y., Li Q., Zhang J., Huang Q. (2014). Effects of biochar amendment on rapeseed and sweet potato yields and water stable aggregate in upland red soil. Catena.

[cit43] Xie J. S., Yang Y. S., Chen G. S., Zhu J. M., Zeng H. D., Yang Z. J. (2008). Effects of vegetation restoration on water stability and organic carbon distribution in aggregates of degraded red soil in subtropics of China. Acta Ecol. Sin..

[cit44] Zheng X. B., Fan J. B., Zhou J. (2015). Effects of Biogas Slurry on Soil Organic Matter and Characteristics of Soil Aggregates in Upland Red Earth. Sci. Agric. Sin..

[cit45] Sharifi Z., Azadi N., Certini G. (2017). Fire and Tillage as Degrading Factors of Soil Structure in Northern Zagros Oak Forest, West Iran. Land Degradation & Development.

[cit46] Zhang P., Jia Z. K., Wang W., Wen-Tao L. U., Gao F., Jun-Feng N. (2012). Effects of Straw Returning on Characteristics of Soil Aggregates in Semi-arid Areas in Southern Ningxia of China. Sci. Agric. Sin..

[cit47] Liu M. Y., Chang Q. R., Qi Y. B. (2006). Fractal characteristics of soil aggregates and micro aggregates in different land use types. Science of Soil and Water Conservation.

[cit48] Li C., Cao Z., Chang J., Zhang Y., Zhu G., Zong N., He Y., Zhang J., He N. (2017). Elevational gradient affect functional fractions of soil organic carbon and aggregates stability in a Tibetan alpine meadow. Catena.

[cit49] Zhu Q. L., Cheng M., Shao-Shan A. N., Xue Z. J. (2013). Effects of Re-vegetation on Characteristics of Soil Aggregates and Humus in Soil Aggregate in Loess Hilly Region of Southern Ningxia. J. Soil Water Conserv..

[cit50] Tamura M., Suseela V., Simpson M., Powell B., Tharayil N. (2017). Plant litter chemistry alters the content and composition of organic carbon associated with soil mineral and aggregate fractions in invaded ecosystems. Global Change Biology.

[cit51] Esch E. H., Lipson D., Cleland E. E. (2017). Direct and indirect effects of shifting rainfall on soil microbial respiration and enzyme activity in a semi-arid system. Plant Soil.

[cit52] Conn C., Dighton J. (2000). Litter quality influences on decomposition, ectomycorrhizal community structure and mycorrhizal root surface acid phosphatase activity. Soil Biol. Biochem..

[cit53] Kozdrój J., Elsas J. D. V. (2000). Response of the bacterial community to root exudates in soil polluted with heavy metals assessed by molecular and cultural approaches. Soil Biol. Biochem..

[cit54] Wang Q. Y., Zhou D. M., Long C. (2009). Microbial and enzyme properties of apple orchard soil as affected by long-term application of copper fungicide. Soil Biol. Biochem..

[cit55] Belyaeva O. N., Haynes R. J., Birukova O. A. (2005). Barley yield and soil microbial and enzyme activities as affected by contamination of two soils with lead, zinc or copper. Biol. Fertil. Soils.

[cit56] Yan J. L. , Distribution, activity and ecological indicator function of enzymes in wetland and paddy soils, Nanjing Agricultural University, 2011

[cit57] Gregory P. J. (2010). Roots, rhizosphere and soil: the route to a better understanding of soil science?. Eur. J. Soil Sci..

[cit58] Eivazi F. (1977). Phosphatase in soils. Soil Biol. Biochem..

[cit59] Page A. L. , Methods of soil analysis. Part 2. Chemical and microbiological properties, Wi American Society of Agronomy Inc & Soil Science Society of America Inc, 1965

